# Multi-Responsive and Antibacterial Dynamic Covalent Hydrogels Cross-Linked by Amphiphilic Copolymer Micelles

**DOI:** 10.3390/gels12010027

**Published:** 2025-12-28

**Authors:** Yuyao Wang, Dou Jin, Zichen Huang, Fan Chen, Kun Liu, Xiacong Zhang

**Affiliations:** Department of Polymer Materials, School of Materials Science and Engineering, Shanghai University, Shanghai 200444, China

**Keywords:** amphiphilic quaternized copolymers, borate ester, dynamic covalent hydrogels, stimuli-responsive polymers

## Abstract

Dynamic covalent hydrogels exhibiting multi-responsive and antibacterial properties offer significant potential for biomedical applications, including smart wound dressings and controlled drug delivery. Herein, a series of amphiphilic quaternized copolymers (Q-C8PEG-n) with tunable quaternization degrees was synthesized from C8PEG via iodomethane addition and characterized by ^1^H NMR, COSY, FTIR, UV-vis spectroscopy, DLS, TEM, and zeta potential analyses, confirming successful quaternization and micelle formation. These copolymers displayed thermosensitive behavior, with cloud point temperatures increasing due to enhanced hydrophilicity. Q-C8PEG-3 micelles, incorporating diethanolamine units, were crosslinked with phenylboronic acid-grafted hyaluronic acid (HA-PBA) to yield dynamic covalent hydrogels (Gel) through reversible boronic ester bonds stabilized by B-N coordination. The Gel exhibited multi-responsiveness, undergoing degradation in acidic or alkaline conditions and exposure to glucose or H_2_O_2_. SEM confirmed a porous microstructure, enabling efficient drug encapsulation, as demonstrated by the release of Nile red (NR). In vitro antibacterial tests revealed enhanced post-quaternization efficacy, with the Gel showing strong activity against *S. aureus*. This micelle-crosslinked platform synergistically combines tunable stimuli-responsiveness with inherent antibacterial properties, holding promise for applications in wound healing and tissue engineering.

## 1. Introduction

Bacterial infections pose a significant challenge during wound healing, representing a major threat to human health and complicating recovery processes [1]. Conventional wound dressings often exhibit limited functionality, such as a single biological activity, which hinders effective tissue repair and increases the risk of chronic wounds [2]. In response, hydrogels have emerged as promising materials due to their excellent biocompatibility, high water content, tunable porosity, oxygen permeability, and mechanical properties that mimic the extracellular matrix (ECM), making them ideal vehicles for bioactive agent delivery in biomedical applications [3,4]. Natural polymer-based hydrogels, derived from substances like sodium alginate (SA), carboxymethylcellulose (CMC), chitosan (CS), chitin, gelatin, and hyaluronic acid (HA), offer additional advantages including non-toxicity, biodegradability, and inherent bioactivity. When integrated with synthetic polymers or loaded with therapeutic agents, these hydrogels show particular promise as advanced wound dressings [5,6]. Among them, HA-based hydrogels have been extensively explored for tissue regeneration, wound management, and drug delivery owing to HA’s role in promoting cell migration, angiogenesis, and ECM remodeling [7,8,9,10]. Li et al. developed a single-component HA hydrogel crosslinked via phenylboronic ester linkages, achieving gelation through simple pH adjustment for effective hemostasis and wound closure [11].

Traditional hydrogels, reliant on irreversible covalent bonds, struggle to adapt to the dynamic and heterogeneous wound microenvironment, often leading to suboptimal healing outcomes. To address this, incorporating dynamic covalent bonds—such as imine, disulfide, or boronate ester linkages—enables reversible bond breakage and reformation, imparting hydrogels with advanced features like stimuli-responsiveness, self-healing, shape adaptability, and enhanced antibacterial properties. These dynamic systems can respond to physiological cues, facilitating on-demand functionality in complex wound settings [12,13,14]. For example, copolymers like P(DMAA-3-AAPBA), formed from *N*, *N*-dimethylacrylamide (DMAA) and 3-(acrylamido)phenylboronic acid (3-AAPBA), utilize dynamic phenylboronate bonds as crosslinkers to create multi-responsive hydrogels sensitive to temperature, glucose, and fructose. Recent advances have further highlighted boronate ester-crosslinked HA hydrogels for their pH-dependent stability and potential in controlled delivery, though challenges remain in maintaining integrity at physiological pH (~7.4) [15].

Stimuli-responsive hydrogels extend this adaptability by rapidly altering mechanical properties or solubility in response to environmental changes, such as pH shifts [16], light exposure [17] and temperature variations [18,19,20], thereby promoting wound repair and facilitating drug release [21]. For instance, acidic or inflammatory wound sites (pH ~4–6) can trigger bond hydrolysis for targeted therapy, while temperature-responsive systems exploit body heat for gel-sol transitions. Notable examples include thermosensitive PEG-PPG-PEG triblock copolymers for pH-dependent release of Nile red (NR) and irinotecan from micelles [22]; UV-light-crosslinked antibacterial hydrogels from *o*-phthalaldehyde-terminated four-arm polyethylene glycol (4aPEG-OPA) and methacrylated *ε*-poly-L-lysine (*ε*-PLL-MA) for diabetic foot ulcers [23]; injectable ROS-responsive supramolecular hydrogels with micelle-loaded drugs for diabetic wound healing [24]; and thermo-responsive P(NAM-co-DAA) copolymers with ketone groups for controlled release [25]. Integrating antibacterial functionalities, such as quaternary ammonium groups, further combats infection risks, with recent dual-responsive (ROS/pH) hydrogels incorporating amphiphilic micelles showing enhanced efficacy against infected wounds.

In our previous work, where an amphiphilic alternating copolymer (C5PEG) was synthesized and gelled with phenylboronic acid-grafted alginate (Alg-PBA) to produce tunable, thermo-responsive hydrogels via boronic ester formation, this study advances the field by developing multi-responsive and antibacterial dynamic covalent hydrogels crosslinked by amphiphilic copolymer micelles [26]. We also prepared a series of amphiphilic copolymers with various aliphatic amine side chains, and investigated the effects of adjusting the copolymers’ mixing ratio, solution’s pH, and the addition of salts on the polymer’s phase transition temperature [27]. Herein, an amphiphilic copolymer (C8PEG), comprising poly(ethylene glycol) diglycidyl ether (PEGDE) and *n*-octylamine segments, was synthesized. A series of quaternized derivatives (Q-C8PEG-n) was then prepared to form antibacterial micelles, characterized for their transition temperature (Tcp) and critical micelle concentration (CMC). These micelles were subsequently crosslinked with phenylboronic acid-grafted hyaluronic acid (HA-PBA) to yield dynamic covalent hydrogels via boronic ester linkages and B-N coordination. This dual-functional design is intended to develop both a wound coverage material and a drug-loaded platform; it synergizes the dynamic covalent hydrogel with the antibacterial activity of drug-loaded quaternized micelles, offering a novel hydrogel-based platform for advanced wound healing applications.

## 2. Result and Discussion

### 2.1. Synthesis and Characterization of Copolymers

**Figure 1:**
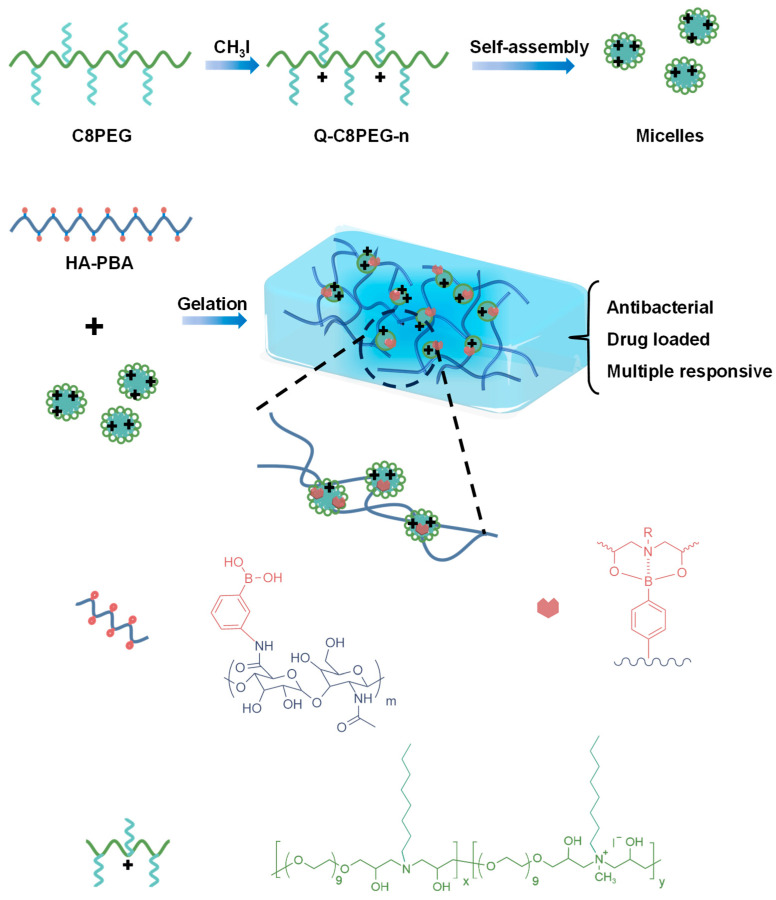
Schematic diagram of the synthesis of dynamic covalent hydrogels cross-linked by amphiphilic copolymer micelles.

**Figure 2:**
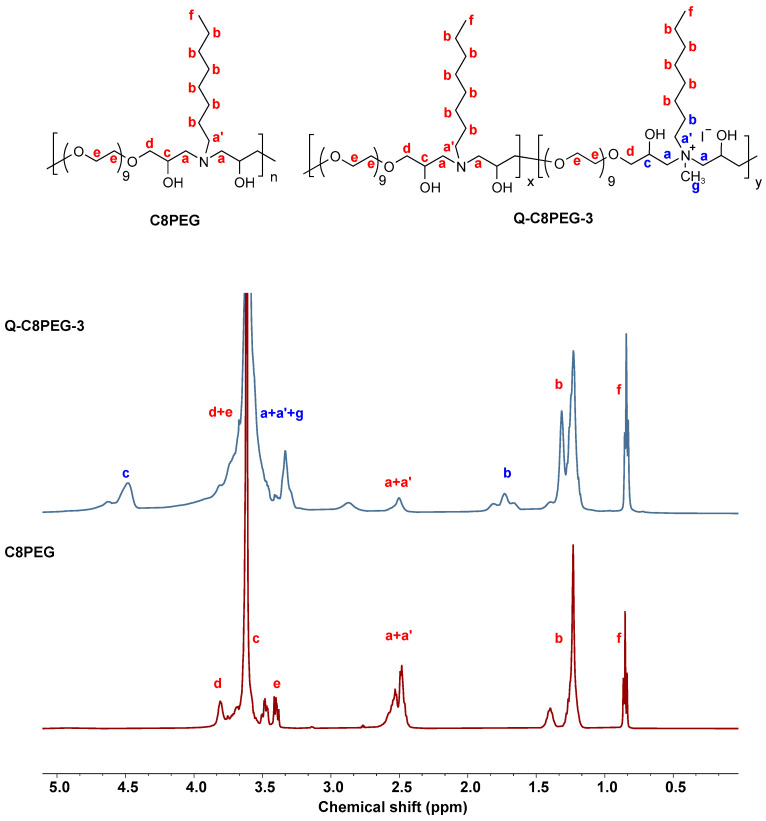
^1^H NMR spectra of C8PEG and Q-C8PEG-3 in CDCl_3_.

C8PEG was synthesized according to our previous work [27], via the amine–epoxy click reaction between poly(ethylene glycol) diglycidyl ether (PEGDE) and *n*-octylamine (C8-NH_2_), yielding a colorless viscous liquid (86%). Subsequently, different concentrations of iodomethane (CH_3_I) were added to the methanol solution of C8PEG, and the mixture was stirred at room temperature for 36 h, after which amphiphilic quaternized copolymers (Q-C8PEG-n) were obtained with yields exceeding 80% (Figure 1). The structural formulas are presented in Figure S1. To confirm the molecular structures, ^1^H NMR characterization was performed on C8PEG, partially quaternized polymers Q-C8PEG-1, Q-C8PEG-2, Q-C8PEG-3, as well as the fully quaternized polymer Q-C8PEG-4 (Figures S2–S6). Comparison of the spectra revealed distinct changes indicative of successful quaternization. To further verify successful quaternization and assign proton couplings, ^1^H-^1^H COSY was employed for C8PEG, Q-C8PEG-2, Q-C8PEG-3, and Q-C8PEG-4. As shown in Figure 2, the chemical shifts of the methylene protons adjacent to the tertiary amine sites (labeled a and a’) shifted downfield to 3.3 ppm after quaternization, reflecting the increased electron withdrawal due to the positively charged quaternary ammonium group. Similarly, the methylene protons on the alkyl chain (b) shifted from 1.4 ppm to 1.7 ppm, consistent with deshielding effects from quaternization. The COSY spectra of Q-C8PEG-2 and Q-C8PEG-3 (Figures S8 and S9) displayed two distinct ^1^H-^1^H coupling signals between a’ and b, confirming the structural integrity of the modified chains. Additionally, the methine proton (c) adjacent to the hydroxyl group in the PEGDE segment shifted from 3.4 ppm to 4.5 ppm post-quaternization, with coupling signals between c and a observed in the COSY spectra of Q-C8PEG-2, Q-C8PEG-3, and Q-C8PEG-4 (Figures S8–S10). Notably, as the degree of quaternization increased, the integral area of the signal for proton c also grew, providing quantitative evidence of progressive modification. These NMR and COSY results collectively demonstrate the successful and tunable quaternization of C8PEG, enabling control over the amphiphilic balance in the copolymers.

**Figure 3:**
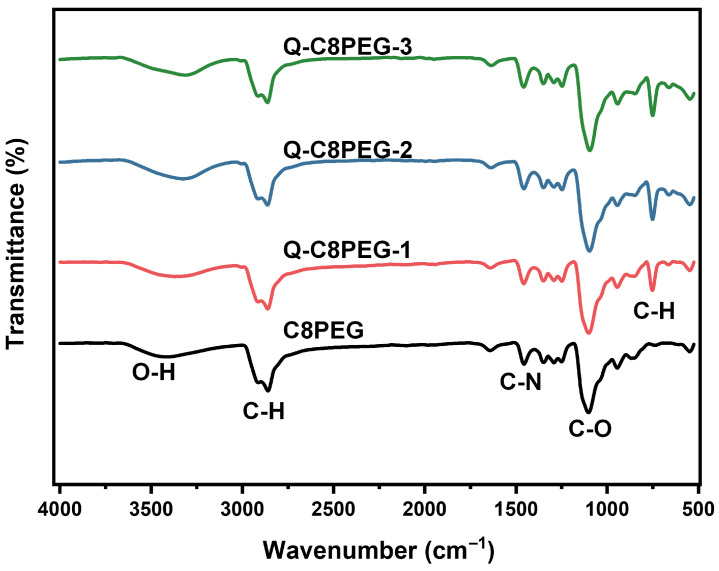
ATR-FTIR spectra of C8PEG, Q-C8PEG-1, Q-C8PEG-2 and Q-C8PEG-3.

The molecular structures of the Q-C8PEG-n copolymers were further corroborated by FTIR spectroscopy. As shown in Figure 3, based on our previous research, the broad peak at approximately 2860 cm^−1^ corresponds to the stretching vibrations of alkyl C-H bonds, and the strong absorption band at 1094 cm^−1^ is attributed to C-O stretching vibrations. Differently with C8PEG, the peak at 750 cm^−1^ in Q-C8PEG-n is attributed to the out-of-plane rocking vibration of the methylene (CH_2_) groups in the alkyl chains. This peak arises from the quaternization-induced conformational ordering of the chains [28]. Q-C8PEG-n showed an enhancement in the peaks related to N-CH_3_ at 1462 cm^−1^, further confirming the successful introduction of CH_3_I [29]. These spectral changes not only validate the chemical modifications but also highlight how quaternization enhances the cationic character of the copolymers, which is crucial for their self-assembly into micelles.

**Figure 4:**
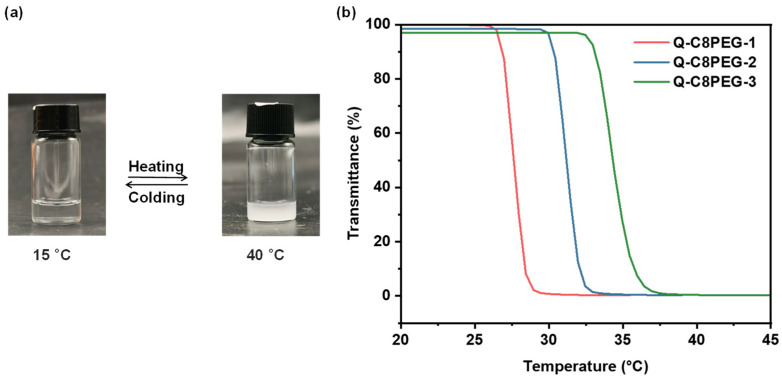
(**a**) Photographs of an aqueous solution of Q-C8PEG-2 at 15 °C (**left**) and 40 °C (**right**). (**b**) The curves of transmittance versus temperature for Q-C8PEG-1, Q-C8PEG-2, and Q-C8PEG-3 in the 3 wt% aqueous solution.

The thermoresponsiveness of the Q-C8PEG-n copolymers in aqueous solution is illustrated in Figure 4a. At low temperatures, the polymer chains remain well-hydrated and dispersed in water, yielding a clear and transparent solution. As the temperature increases to the cloud point temperature (Tcp), the chains undergo dehydration and hydrophobic aggregation, resulting in solution turbidity and demonstrating reversible thermoresponsive behavior characteristic of lower critical solution temperature (LCST)-type polymers. To quantify these thermoresponsive properties, the transmittance of 3 wt% Q-C8PEG-n aqueous solutions at 700 nm was measured using variable-temperature UV-vis spectroscopy. Notably, the phase transition temperature of the non-quaternized C8PEG could not be determined, as it remained turbid even in an ice-water bath at 0 °C, indicating an LCST below this temperature due to its higher hydrophobicity.

As depicted in Figure 4b, the quaternized Q-C8PEG-n copolymers exhibit enhanced hydrophilicity, rendering them clear and transparent at room temperature. Upon heating to their respective Tcp values, the solutions become turbid, with a sharp drop in transmittance. The measured Tcp values were 27.6 °C for Q-C8PEG-1, 31.3 °C for Q-C8PEG-2, and 34.4 °C for Q-C8PEG-3. This increasing trend in Tcp correlates with higher quaternization degrees, which elevate charge density and hydrophilicity, thereby requiring greater thermal energy to disrupt polymer-water interactions and induce phase separation. For Q-C8PEG-4 (fully quaternized), no phase transition was observed up to 60 °C, suggesting its Tcp exceeds practical measurement ranges due to maximal ionic hydration. Cooling the solutions back to room temperature restored clarity and transparency, confirming the reversibility of the temperature-induced phase transition. These findings align with literature on cationic thermoresponsive polymers, where quaternization enhances solubility and tunes Tcp by modulating electrostatic repulsions and hydration shells, which is critical for applications in stimuli-responsive hydrogels.

To further investigate the aggregation behavior of the polymers, the hydrophobic dye 1, 6-diphenyl-1, 3, 5-hexatriene (DPH) was employed as a fluorescent probe to determine the critical micelle concentration (CMC) of the Q-C8PEG-n copolymers [22]. Above the CMC, the formation of hydrophobic micelle cores enables the encapsulation of DPH, resulting in a marked increase in its absorbance due to the dye’s enhanced solubility in the nonpolar environment. Conversely, below the CMC, DPH remains poorly solubilized in the aqueous phase, yielding low absorbance signals. The CMC values for C8PEG and Q-C8PEG-n were determined by plotting the absorbance at 377–400 nm against the logarithm of the polymer concentration and identifying the inflection point via linear fitting.

**Figure 5:**
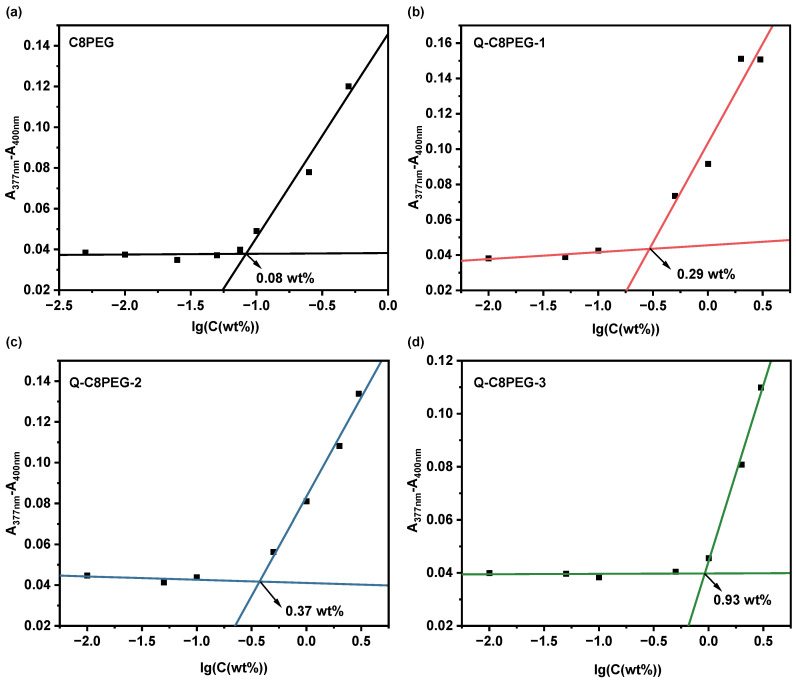
Absorption of the hydrophobic probe DPH in (**a**) C8PEG, (**b**) Q-C8PEG-1, (**c**) Q-C8PEG-2, and (**d**) Q-C8PEG-3 polymer solutions was tested at 25 °C, with the CMC of each polymer determined by extrapolation of the absorbance difference at 377 and 400 nm.

As shown in Figure 5, the CMC values were 0.08 wt% for C8PEG, 0.29 wt% for Q-C8PEG-1, 0.37 wt% for Q-C8PEG-2, and 0.93 wt% for Q-C8PEG-3. Notably, no micelle formation was observed for Q-C8PEG-4 even at concentrations up to 5 wt%, indicating that full quaternization imparts excessive hydrophilicity, preventing self-assembly under these conditions. The observed increase in CMC with quaternization degree reflects the enhanced ionic character and hydration of the copolymers, which strengthens polymer-water interactions and raises the energy barrier for hydrophobic aggregation. This trend is consistent with the previously noted elevation in Tcp (Figure 4b), where greater charge density similarly delays phase separation by promoting solubility.

Above the CMC, at a 1 wt% concentration and 25 °C, dynamic light scattering (DLS) was utilized to assess the hydrodynamic diameters of the micelles formed by Q-C8PEG-1, Q-C8PEG-2, and Q-C8PEG-3. The results revealed average diameters of approximately 70 nm for Q-C8PEG-1, 120 nm for Q-C8PEG-2, and 126 nm for Q-C8PEG-3. This progressive increase in particle size with quaternization degree can be attributed to the formation of a thicker hydration shell around the micelles, driven by the higher density of quaternary ammonium groups, which attract more water molecules and expand the apparent volume. However, the modest size difference between Q-C8PEG-2 and Q-C8PEG-3 suggests a plateauing effect at higher quaternization levels, possibly due to balanced electrostatic repulsions that stabilize looser micellar structures. These micelle characteristics are pivotal for their role as dynamic crosslinkers in the hydrogels, where tunable size and stability influence network formation, responsiveness, and antibacterial efficacy, as quaternized groups confer cationic surfaces that disrupt bacterial membranes.

**Figure 6:**
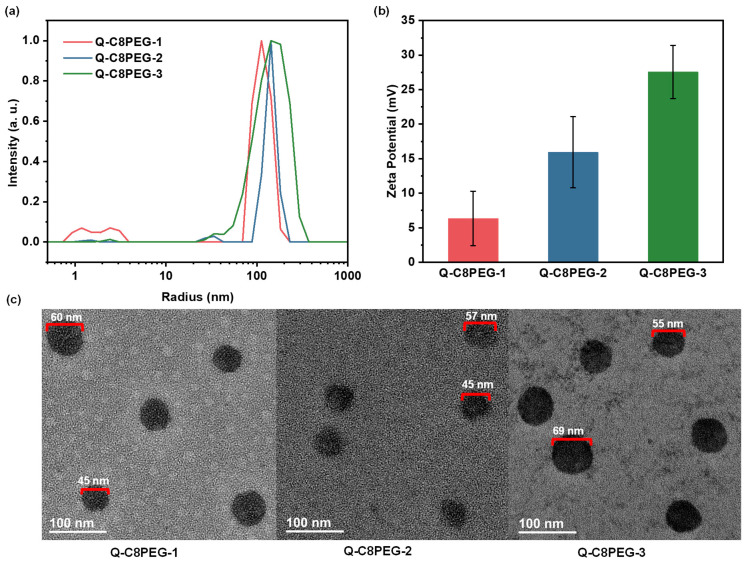
The histogram size distribution (**a**), zeta potential (**b**), and TEM image (**c**) of Q-C8PEG-1, Q-C8PEG-2, and Q-C8PEG-3 micelles.

To complement the DLS measurements, transmission electron microscopy (TEM) was employed to visualize the morphology and dry-state sizes of the micelles. As shown in Figure 6, the TEM images of micelles formed by Q-C8PEG-1, Q-C8PEG-2, and Q-C8PEG-3 revealed spherical structures with diameters in the range of 50–70 nm, which are consistently smaller than the hydrodynamic diameters obtained from DLS (70–126 nm). This size discrepancy is typical for hydrated soft nanomaterials and can be attributed to the evaporation of the surrounding hydration layer during TEM sample preparation, coupled with the low electron contrast of the extended PEG segments in the micellar corona.

Furthermore, zeta potential measurements were conducted to evaluate the surface charge of the micelles in aqueous dispersion (1 wt%, 25 °C). The values were determined to be +6.35 mV for Q-C8PEG-1, +15.9 mV for Q-C8PEG-2, and +27.6 mV for Q-C8PEG-3, exhibiting a clear positive correlation with the degree of quaternization. This trend quantitatively confirms the progressive incorporation of quaternary ammonium groups, which impart increasing cationic character to the micellar surfaces. Positive zeta potentials in this range indicate moderate to good colloidal stability through electrostatic repulsion, preventing aggregation in solution. Importantly, the enhanced positive charge density is key to the antibacterial properties of these micelles, as it promotes strong electrostatic interactions with negatively charged bacterial cell membranes (e.g., in Gram-positive and Gram-negative bacteria), leading to membrane permeabilization, leakage of cellular contents, and ultimately microbial death. These characteristics position the Q-C8PEG-n micelles as effective dynamic crosslinkers in the multi-responsive hydrogels, where tunable charge and size enable pH- or temperature-triggered disassembly, alongside inherent antimicrobial activity to prevent infections in biomedical applications.

To further evaluate the self-assembly capability of the prepared copolymers and their potential as carriers for hydrophobic payloads, Nile red—a hydrophobic dye commonly used as a model for poorly water-soluble drugs—was employed to assess the drug loading content (*DLC*) and encapsulation efficiency (*EE*) in Q-C8PEG-3 micelles. Due to the hydrophobic polycyclic aromatic hydrocarbon structure of Nile red, it can be encapsulated within the hydrophobic core of the micelle through physical interactions. Q-C8PEG-3 was selected for this study due to its balanced amphiphilicity, exhibiting a suitable CMC (0.93 wt%) and micelle size (~126 nm) that facilitate stable encapsulation without excessive hydrophilicity that might hinder core formation, as observed in Q-C8PEG-4. The encapsulation was performed by dissolving Nile red in a polymer solution above the CMC, followed by dialysis to remove unencapsulated dye. The standard curve of Nile red in pH 7.4 PBS solution was expressed by the equation, Y = 0.05176X + 0.08389, where X was the concentration of the Nile red solution in μg/mL and Y was the fluorescence intensity of Nile red. The R^2^ value of the standard curve was 0.99875, which is greater than 0.99, indicating that the regression line fits the data points. The *DLC* and *EE* of Nile red in the Q-C8PEG-3 micelles were around 0.42% and 26.78%.

### 2.2. Synthesis and Characterization of Hydrogel

Hyaluronic acid (HA), a naturally occurring polysaccharide renowned for its excellent biocompatibility, biodegradability, and high water retention capacity, is widely utilized in biomedical applications such as drug delivery and tissue regeneration. Leveraging the dynamic covalent chemistry of boronic ester bonds, which form between phenylboronic acid and diols and can be stabilized by intramolecular B-N coordination for enhanced stability at physiological pH, we synthesized a copolymer of HA and 3-aminophenylboronic acid (3-APBA), denoted as HA-PBA [26]. This copolymer was prepared via carbodiimide-mediated coupling of the carboxylic acid groups on HA with the amine functionality of 3-APBA, following established protocols, and formulated as a 5 wt% stock solution in PBS (pH 7.4) for subsequent hydrogel formation with the quaternized copolymers.

The hydrogel network relies on the reversible boronic ester crosslinks between the PBA moieties on HA-PBA and the diethanolamine units in Q-C8PEG-n, where the nitrogen atom facilitates B-N coordination to lower the pKa of the boronic acid and promote bond formation under neutral conditions. However, experiments revealed that the fully quaternized Q-C8PEG-4 failed to form a stable gel with HA-PBA, as the high density of positive charges likely induces electrostatic repulsion that disrupts the B-N coordination and hinders effective crosslinking between phenylboronic acid and diols. Consequently, Q-C8PEG-3—the copolymer with the highest quaternization degree that still permits micelle formation and balanced charge density—was selected to fabricate the hydrogel sample (denoted as Gel) with HA-PBA for further characterization. This selection ensures optimal amphiphilicity for micelle-mediated crosslinking while maintaining sufficient cationic sites for antibacterial activity.

**Figure 7:**
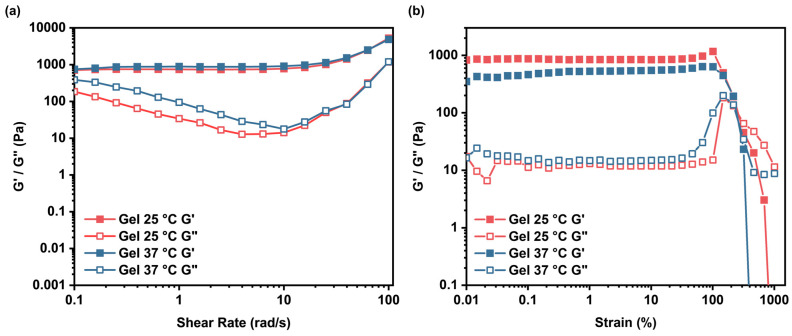
Rheological behavior of Gel at 25 °C and 37 °C (**a**,**b**).

To assess the mechanical properties of the Gel, rheological measurements were conducted using a rotational rheometer. Strain amplitude sweeps (at a fixed angular frequency of 10 rad/s) and angular frequency sweeps (at a fixed strain amplitude of 0.1%) were performed at both 25 °C and 37 °C. As shown in Figure 7, the storage modulus (G’) dominated the loss modulus (G’’) across the tested ranges at both temperatures, confirming the viscoelastic solid-like behavior characteristic of a crosslinked hydrogel network. Notably, at 37 °C, G’ exhibited a slight decrease compared to 25 °C (e.g., from ≈870 Pa to ≈460 Pa at low strain), indicating partial disruption of the internal structure due to temperature-induced weakening of the dynamic crosslinks. This thermosensitivity aligns with the LCST behavior observed in Q-C8PEG-3 (Tcp ≈ 34.4 °C), where elevated temperatures promote hydrophobic collapse of the micellar cores, leading to network softening.

**Figure 8:**
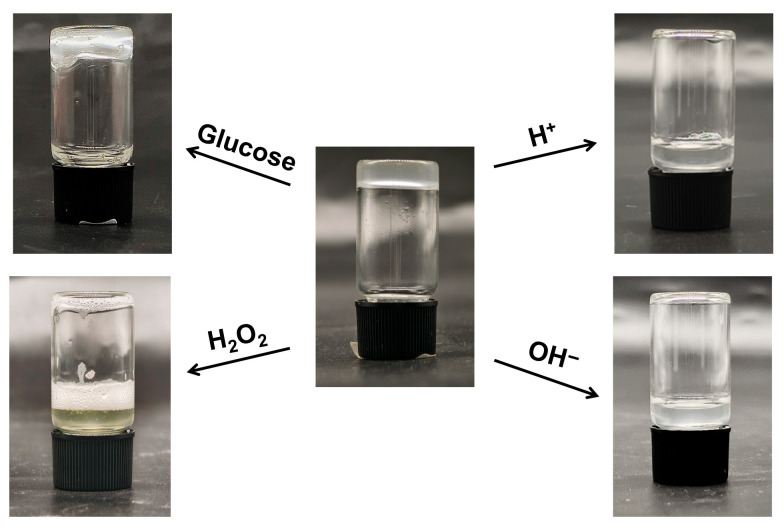
The multi-responsive behavior of Gel.

The multi-responsive nature of the hydrogel was evaluated by assessing its disruption and degradation behavior under various stimuli, focusing on pH (acidic and alkaline conditions) and glucose exposure, which are relevant to physiological environments such as wound sites or diabetic settings where pH fluctuations or elevated glucose levels may trigger controlled responses. The dynamic covalent boronic ester crosslinks in the Gel are inherently sensitive to these stimuli: in acidic media, protonation promotes hydrolysis of the boronic ester bonds, while in alkaline conditions, excess hydroxide ions can facilitate nucleophilic attack and bond cleavage. Glucose, as a competing diol, can displace the diethanolamine units from the boronic acid moieties, potentially leading to network disassembly. As shown in Figure 8, immersion of the Gel in acidic and alkaline solutions resulted in complete degradation within 0.5 h, transforming the hydrogel into a turbid sol state due to the rapid hydrolysis of crosslinks. This swift response highlights the pH-sensitivity of the boronic ester network, enabled by the intramolecular B-N coordination in Q-C8PEG-3, which stabilizes bonds at neutral pH but allows facile dissociation under extreme pH shifts. When the hydrogel was immersed in a 3% H_2_O_2_ solution, it completely degraded within 0.5 h, accompanied by the solution turning yellow and vigorous gas release. This phenomenon arises from the redox reaction between I^−^associated with the quaternized copolymer and H_2_O_2_, which generates I_2_ and breaks the boronic ester. In turn, the I_2_ acts as a catalyst to promote the decomposition of excess H_2_O_2_, resulting in the production of O_2_. In contrast, exposure to a 30 mg/mL glucose solution (pH 7.4) for 0.5 h induced only minor structural weakening, manifesting as slight flow upon vial inversion without full degradation. This limited response may stem from the high stability of the B-N-coordinated boronic esters, requiring higher glucose concentrations or prolonged exposure for competitive binding to fully disrupt the network—consistent with tunable glucose sensitivity in similar systems. These behaviors underscore the hydrogel’s potential for targeted applications, such as pH-triggered drug release in acidic tumor microenvironments or controlled softening in glucose-rich diabetic wounds, while the antibacterial quaternized micelles provide an additional layer of infection resistance. The stability of the hydrogel was further evaluated by immersing it in 1 mL of various aqueous solutions, such as deionized water, PBS solution, 30 mg/mL glucose solution, and ethanol-water mixtures, at room temperature for 24 h. The results showed that the hydrogel only underwent swelling but did not dissolve (Figure S12), demonstrating excellent water retention capacity and structural stability. This characteristic enables the hydrogel to efficiently absorb exudates from wound tissues and maintain a moist wound microenvironment, which is critical for facilitating wound healing.

**Figure 9:**
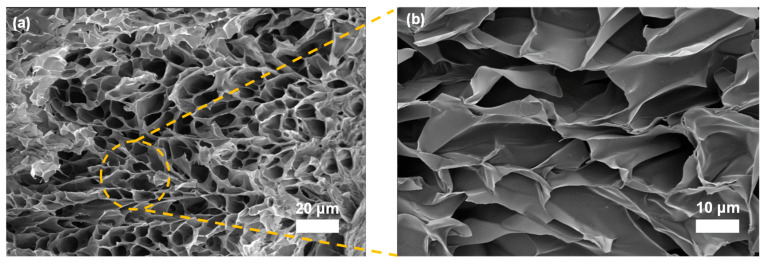
SEM image of Gel (**a**) and magnified image (**b**).

The microstructure of the lyophilized Gel was characterized using scanning electron microscopy (SEM) to elucidate its internal architecture and suitability for biomedical uses. Figure 9a, b depict the overall and magnified views, respectively, revealing a three-dimensional interconnected porous network with pore sizes predominantly in the 8–15 µm range. This porosity arises from the phase separation during micelle-mediated crosslinking and subsequent freeze-drying, where the amphiphilic Q-C8PEG-3 micelles act as templates, creating voids upon solvent sublimation. Such a microporous structure facilitates efficient nutrient diffusion, waste removal, and cell infiltration, making it ideal for tissue engineering scaffolds.

### 2.3. In Vitro Antibacterial Properties

**Figure 10:**
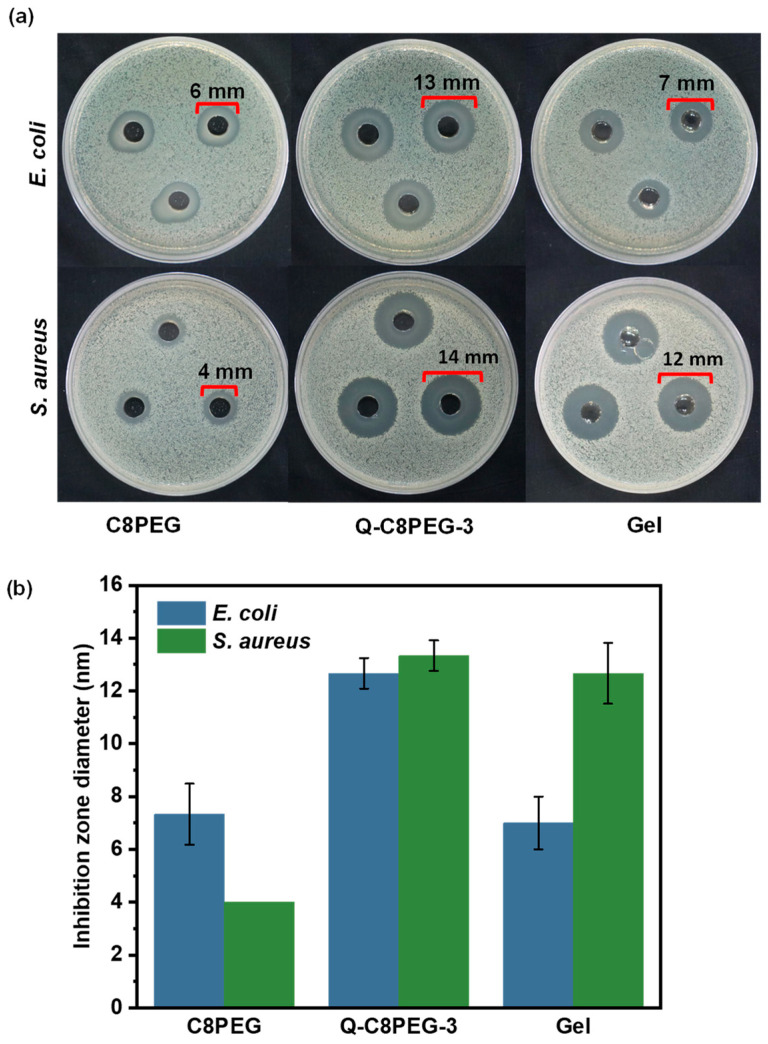
Photographs and quantitative statistics of the inhibition zone against *S. aureus* and *E. coli* in the zone of inhibition test (**a**,**b**).

Quaternized copolymers exhibit strong affinity for negatively charged bacterial cell surfaces through electrostatic interactions, leading to membrane disruption, leakage of intracellular contents, and eventual cell death. This mechanism is particularly effective against both Gram-negative (e.g., *Escherichia coli*, *E. coli*) and Gram-positive (e.g., *Staphylococcus aureus*, *S. aureus*) bacteria, though Gram-negative species often show slightly higher resistance due to their outer lipopolysaccharide membrane, which can impede cationic penetration [30]. To verify the enhancement in antimicrobial performance post-quaternization, the antibacterial efficacies of C8PEG and Q-C8PEG-3 polymer solutions (at equivalent concentrations, e.g., 1 wt%) were compared against *E. coli* and *S. aureus* using the agar disk diffusion (inhibition zone) method. As depicted in Figure 10, the inhibition zones expanded from 6 mm to 13 mm for *E. coli* and from 4 mm to 14 mm for *S. aureus*, demonstrating a substantial improvement in antibacterial activity attributable to the introduction of quaternary ammonium groups, which amplify cationic charge density and membrane-disrupting potential. This enhancement aligns with zeta potential measurements, where Q-C8PEG-3 exhibited a higher surface charge, facilitating stronger electrostatic binding to bacterial surfaces compared to the neutral or weakly charged C8PEG.

When incorporated into the hydrogel, the antibacterial efficacy was evaluated under identical conditions. The inhibition zone against *E. coli* was smaller for the Gel than for the Q-C8PEG-3 solution (e.g., ≈10 mm vs. 13 mm), likely due to slower diffusion of the quaternized micellar crosslinkers from the crosslinked matrix, which restricts their availability at the agar interface. In contrast, the Gel maintained robust efficacy against *S. aureus* (≈14 mm, comparable to the solution), suggesting that the Gram-positive bacterium’s more permeable peptidoglycan wall allows effective interaction even with matrix-bound antimicrobials. These results highlight the hydrogel’s selective antibacterial profile, with potential implications for applications in wound dressings where *S. aureus* is a prevalent pathogen. Overall, the quaternization not only boosts inherent antimicrobial activity but also synergizes with the dynamic covalent network to provide sustained release and localized action, reducing the risk of systemic toxicity while enhancing biocompatibility for tissue engineering.

### 2.4. In Vitro Release of Nile Red from Gel

**Figure 11:**
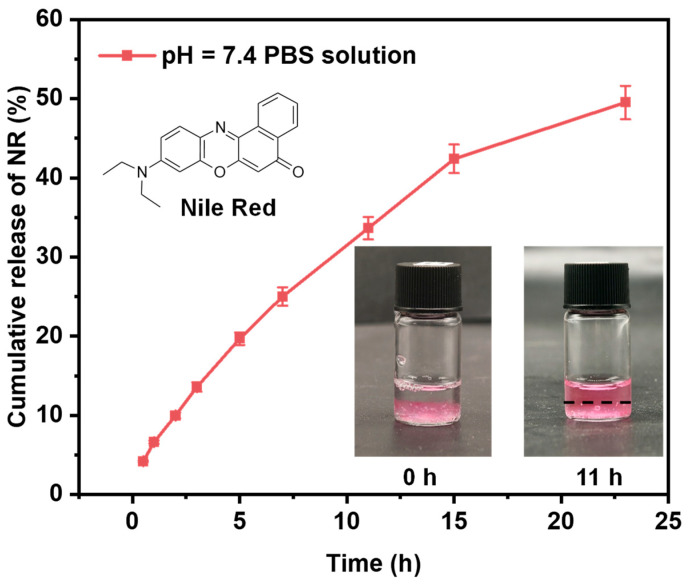
Release profile of Nile red enveloped Gel in PBS solution and the chemical structure of Nile red. (The dashed lines represent the top position of the Gel).

To evaluate the potential of the multi-responsive hydrogel as a drug delivery platform, the in vitro release profile of Nile red (NR)—a hydrophobic dye encapsulated within the Q-C8PEG-3 micelles—was monitored from the Gel over 24 h under physiological conditions. As illustrated in Figure 11, the cumulative release of NR reached approximately 52% at pH 7.4 after 24 h. The release kinetics can be delineated into two distinct phases: an initial burst release (0–15 h), characterized by rapid NR efflux, followed by a sustained slow-release phase (15–24 h). During the burst phase, the primary drivers include the steep concentration gradient of NR across the hydrogel-solvent interface and the initial structural relaxation of the swollen network, facilitating diffusion from the micellar cores through the porous matrix (pore sizes 8–15 µm, Figure 9). In the subsequent slow phase, escalating diffusion resistance—arising from depleted NR reservoirs in peripheral micelles and potential pore occlusion by aggregated components—dominates, with minor contributions from gradual hydrogel degradation under these mild conditions.

## 3. Conclusions

In this study, a series of amphiphilic quaternized copolymers (Q-C8PEG-n) with tunable quaternization degrees was successfully synthesized from the copolymer C8PEG and comprehensively characterized. Key findings revealed clear correlations between quaternization degree and thermoresponsive properties, with cloud point temperatures (Tcp) increasing from 27.6 °C to 34.4 °C as hydrophilicity enhanced, alongside a marked rise in critical micelle concentration (CMC) from 0.08 wt% for C8PEG to 0.93 wt% for Q-C8PEG-3, reflecting diminished self-assembly propensity at higher charge densities. These micelles, particularly those from Q-C8PEG-3, served as effective dynamic crosslinkers when combined with HA-PBA, forming hydrogels via reversible boronic ester bonds stabilized by intramolecular B-N coordination. Rheological analyses confirmed the Gel’s viscoelastic nature and thermoresponsiveness, with a slight softening at physiological temperatures (37 °C) due to micellar aggregation above Tcp. The hydrogel exhibited multi-responsive behavior, degrading rapidly under extreme pH conditions (acidic or alkaline) while showing limited response to glucose, attributable to the stability of the coordinated crosslinks. SEM imaging disclosed a porous microstructure (pore sizes 8–15 µm), ideal for drug encapsulation and diffusion, as evidenced by the biphasic release of Nile red (NR) as a model hydrophobic drug, achieving ~52% cumulative release over 24 h at neutral pH. Furthermore, in vitro antibacterial assays demonstrated enhanced efficacy post-quaternization, with the Gel maintaining strong activity against *S. aureus* (inhibition zones ~14 mm), while showing moderate effects on *E. coli*. Nevertheless, a comprehensive evaluation of the hydrogel’s biocompatibility—via cytotoxicity assays and histological analyses—will be required in subsequent studies.

Overall, this work presents a versatile platform for multi-responsive and antibacterial hydrogels, where amphiphilic micellar crosslinkers enable tunable stimuli-sensitivity (temperature, pH, glucose, and H_2_O_2_) and inherent antimicrobial properties, holding significant promise for biomedical applications such as smart wound dressings, tissue engineering scaffolds, and controlled drug delivery systems. Future investigations could explore in vivo biocompatibility, optimization of quaternization for broader antibacterial spectra, and integration of therapeutic payloads to advance clinical translation.

## 4. Materials and Methods

### 4.1. Materials

Poly(ethylene glycol) diglycidyl ether (PEGDE; average molecular weight = 500) and *n*-octylamine (C8-NH_2_, > 98%) were purchased from Aladdin (Shanghai, China). Iodomethane (CH_3_I) was supplied by Adamas-beta (Shanghai, China). Hyaluronic acid (HA) and 3-Aminobenzeneboronic acid (3APBA) were purchased from Macklin (Shanghai, China).

### 4.2. Experimental Section

#### 4.2.1. Synthesis of C8PEG

Generally, PEGDE (2.0 g, 3.8 mmol) and C8-NH_2_ (628 µL, 3.8 mmol) were added to a 25 mL Schlenk tube. The ethanol was also added as a solvent. The air in the tube should be replaced with N_2_. After stirring for 12 h at 60 °C, ethanol was removed by rotary evaporation. The product was dissolved in dichloromethane (DCM), and washed with a 10 wt% NaOH aqueous solution three times to remove the unreacted monomers. After removing the DCM via rotary evaporation, a colorless, viscous liquid was obtained ultimately.

#### 4.2.2. Synthesis of Q-C8PEG-n

Q-C8PEG-n with different quaternization degrees were prepared by adjusting the molar ratios of C8PEG to iodomethane. Particularly, C8PEG (1 g, 1.5 mmol) was dissolved in 8 mL of methanol, and 96 μL (1.5 mmol), 192 μL (3.0 mmol), 384 μL (6.0 mmol) CH_3_I were added to synthesize the Q-C8PEG-1, Q-C8PEG-2, Q-C8PEG-3, respectively. It should be noted that Q-C8PEG-4 was synthesized by adding excess CH_3_I to ensure the completion of the quaternization reaction at C8PEG polymers. The mixture was stirred at room temperature for 36 h, after which methanol was removed via rotary evaporation, and the colorless viscous liquid was obtained.

### 4.3. Characterization of Synthesized Polymers

^1^H NMR (in CDCl_3_) spectra of C8PEG and Q-C8PEG-n were performed on an NMR spectrometer (AV-500; Bruker, Zurich, Switzerland) to confirm the successful synthesis of quaternization. Correlation spectroscopy (COSY) spectra are obtained to reveal the couplings between protons. Fourier-transform infrared (FTIR) spectroscopies of polymers were obtained using an FTIR spectrometer (Nicolet iS 50; Thermo Fisher Scientific, Waltham, MA, USA).

### 4.4. Characterization of the Self-Assembly Process of Polymer Micelles

The hydrodynamic radius of Q-C8PEG-n micelles was measured using a dynamic light scattering (DLS) instrument (DynaPro NanoStar M3300; Wyatt, Santa Barbara, CA, USA). Before measurement, all the solutions were filtered through a 0.8 µm syringe filter to remove dust. The zeta potential of the prepared Q-C8PEG-n solutions was determined using a Zetasizer Nano-ZS90 (Malvern Instruments Ltd., Malvern, UK). Samples were deposited onto a copper grid containing a carbon film, and after the water had completely evaporated, stained with 0.2 mg/mL uranyl acetate, and then the Transmission electron microscopy (TEM) images were obtained from JEM-2100F Transmission Electron Microscope (JEOL Ltd., Tokyo, Japan).

The critical micelle concentrations (CMC) of C8PEG and Q-C8PEG-n in water were measured by the dye solubilization method using 1,6-diphenyl-1,3,5-hexatriene (DPH) as a hydrophobic dye probe [22]. Aqueous solutions of C8PEG (1 mL) at varying concentrations (0.005, 0.01, 0.025, 0.05, 0.075, 0.1, 0.25, and 0.5%) and Q-C8PEG-n at varying concentrations (0.01, 0.05, 0.1, 0.5, 1, 2, and 3 wt%) were prepared, into each of which 10 μL of 0.4 mM DPH methanol solution was added. These mixture solutions were equilibrated in the dark overnight at 4 °C before their absorbance from 300 to 450 nm was measured via a Microplate reader (Multiskan SkyHigh, Thermo Fisher Scientific, Vantaa, Finland). The inflection points of the absorbance difference between 377 and 400 nm (A_377nm_–A_400nm_) against the logarithmic concentration of the polymer solution were equal to the CMC value.

A UV–vis spectrophotometer (V750; JASCO, Hachioji, Tokyo, Japan) equipped with a thermo-controlled bath was utilized to analyze the polymer tunable cloud points (Tcp) temperature of Q-C8PEG-n solutions at a concentration of 3 wt%. The sample was heated at a constant rate of 0.5 °C·min^−1^, and absorbance was recorded every 5 s.

### 4.5. Nile Red-Loaded Q-C8PEG-n Micelles

Briefly, 50 mg of Q-C8PEG-3 was dissolved in 1 mL of Nile red (NR) solution in THF (1 mg/mL). Then, 0.5 mL phosphate-buffered saline (PBS) buffer was added, and the mixture was stirred in the dark overnight to obtain NR-loaded micelles, which subsequently were dialyzed (MWCO = 3500 Da) against PBS solution (pH = 7.4) containing 0.5% Tween 80 for 24 h to remove unloaded NR and THF. After dialysis, the concentration of NR inside the dialysis tube was measured to determine its loading capacity after being diluted with PBS solution. The drug loading content (*DLC*) and the encapsulation efficiency (*EE*) were determined according to the following formulae:

$$DLC\%=\frac{W_{loaded\;drug}}{W_{micelles}+W_{loaded\;drug}}$$

$$EE\%=\frac{W_{loaded\;drug}}{W_{free\;drug}}$$

### 4.6. Formation and Characterization of the Hydrogels

HA-PBA, synthesized according to Jin’s work, was dissolved in water with a concentration of 5 wt% [31]. Q-C8PEG-3 (400 μL, 3 wt%) and 256 μL HA-PBA solution were mixed to form Gel. 

The resulting gel was cut into pieces after being lyophilized, sputter-coated with a platinum layer, and mounted on iron specimen mounts using carbon adhesive tabs. The internal morphology of the hydrogel was then characterized using a field-emission scanning electron microscope (Gemini SEM 300, Carl Zeiss AG, Oberkochen, Germany).

The rheology of Gel was conducted by rheometers (MCR302, Anton Paar Instruments, Graz, Austria) at 25 °C and 37 °C. The storage modulus (G′) and loss modulus (G″) of hydrogels were studied using a frequency-sweep mode between 0.1 and 100 rad/s with 0.1% strain. The strain amplitude sweep was scanned between 0.01% and 1000% with 10 rad/s. Temperature sweep was conducted from 20 °C to 80 °C of a constant rate of 0.5 °C·min^−1^ at a fixed frequency of 10 rad/s and fixed strain amplitude of 0.1%.

### 4.7. Multi-Responsive Behaviors and Stability of the Hydrogels

In the glass bottles containing 625 μL gel, 1 mL of 0.3% HCl aqueous solution, 1% NaOH aqueous solution 3% H_2_O_2_ solution, and 30 mg/mL glucose solution were added for soaking. After 0.5 h, the sample bottles were inverted to observe the gel state.

The stability of the hydrogel was further evaluated by immersing it in 1 mL of deionized water, PBS solution, 30 mg/mL glucose solution, and 50% ethanol-water mixtures (*v*/*v*), at room temperature for 24 h.

### 4.8. In Vitro Release of Nile Red from Gel

60 μL of NR solution in THF (1 mg/mL) and 1 mL Q-C8PEG-n aqueous solution were mixed and incubated in the dark for 24 h in an open container to evaporate the THF. Then, the mixture solution was added to the HA-PBA aqueous solution to obtain a Gel as in the former methods. The gel was immersed in 1 mL PBS (pH = 7.4) solution and stirred at 37 °C. The release medium was all collected and replaced with 1 mL of new PBS solution at predetermined time points. The drug concentrations of loaded drugs were determined by a Microplate Reader at 550 nm.

### 4.9. In Vitro Antibacterial Properties of the Q-C8PEG-3 Solutions and Hydrogels

The bacterial colonies of Escherichia coli (*E. coli*) and Staphylococcus aureus (*S. aureus*) were inoculated into sterile test tubes containing 3–5 mL of liquid Luria–Bertani (LB) medium, and then placed in a shaking incubator at 37 °C (200–250 rpm) for 12–16 h until the bacterial solution becomes turbid (OD_600nm_ ≈ 2.0–4.0). Dilute the bacterial solution with PBS to 10^6^ CFU/mL, and take 100 μL of the diluted solution to spread it on a plate. Use a puncher to make holes, in each of which 100 μL of 3 wt% Q-C8PEG-3 solution (ultrasonically for 30 min before adding) was added, and attach a piece of gel (625 μL) to the surface of the plate. Place them all in a 37 °C constant temperature incubator for static cultivation for 24 h.

## Data Availability

The original contributions presented in this study are included in the article/Supplementary Materials. Further inquiries can be directed to the corresponding authors.

## Supplementary Materials

The following supporting information can be downloaded at: https://www.mdpi.com/article/10.3390/gels12010027/s1, Figure S1: C8PEG and Q-C8PEG-n copolymers synthesis and the gelation with HA-PBA; Figure S2: ^1^H NMR spectrum of C8PEG in CDCl3; Figure S3: ^1^H NMR spectrum of Q-C8PEG-1 in CDCl_3_; Figure S4: ^1^H NMR spectrum of Q-C8PEG-2 in CDCl_3_; Figure S5: ^1^H NMR spectrum of Q-C8PEG-3 in CDCl_3_; Figure S6: ^1^H NMR spectrum of Q-C8PEG-4 in CDCl_3_; Figure S7: ^1^H-^1^H NMR COSY spectrum of C8PEG in CDCl_3_; Figure S8: ^1^H-^1^H NMR COSY spectrum of Q-C8PEG-2 in CDCl_3_; Figure S9: ^1^H-^1^H NMR COSY spectrum of Q-C8PEG-3 in CDCl_3_; Figure S10 ^1^H-^1^H NMR COSY spectrum of Q-C8PEG-4 in CDCl_3_; Figure S11: Temperature sweep of Gel from 20 °C to 80 °C; Figure S12: The stability assessment of hydrogels in 1 mL PBS solution, glucose, H_2_O, and ethanol-water mixtures.
