# Supplementary material for: Multi-Responsive and Antibacterial Dynamic Covalent Hydrogels Cross-Linked by Amphiphilic Copolymer Micelles
Source: Gels. 2025 Dec 28;12(1):27. doi: 10.3390/gels12010027 (PMC12841037; doi:10.3390/gels12010027)
Supplement: Supplementary file 1 [file gels-12-00027-s001.zip › gels-4046072-supplementary.pdf]

Supplementary Materials

Multi-Responsive and Antibacterial Dynamic Covalent Hydrogels Cross-Linked by Amphiphilic Copolymer Micelles

Yuyao Wang, Dou Jin, Zichen Huang, Fan Chen, Kun Liu [1] and Xiacong Zhang

Department of Polymer Materials, School of Materials Science and Engineering, Shanghai University, Shanghai 200444, China

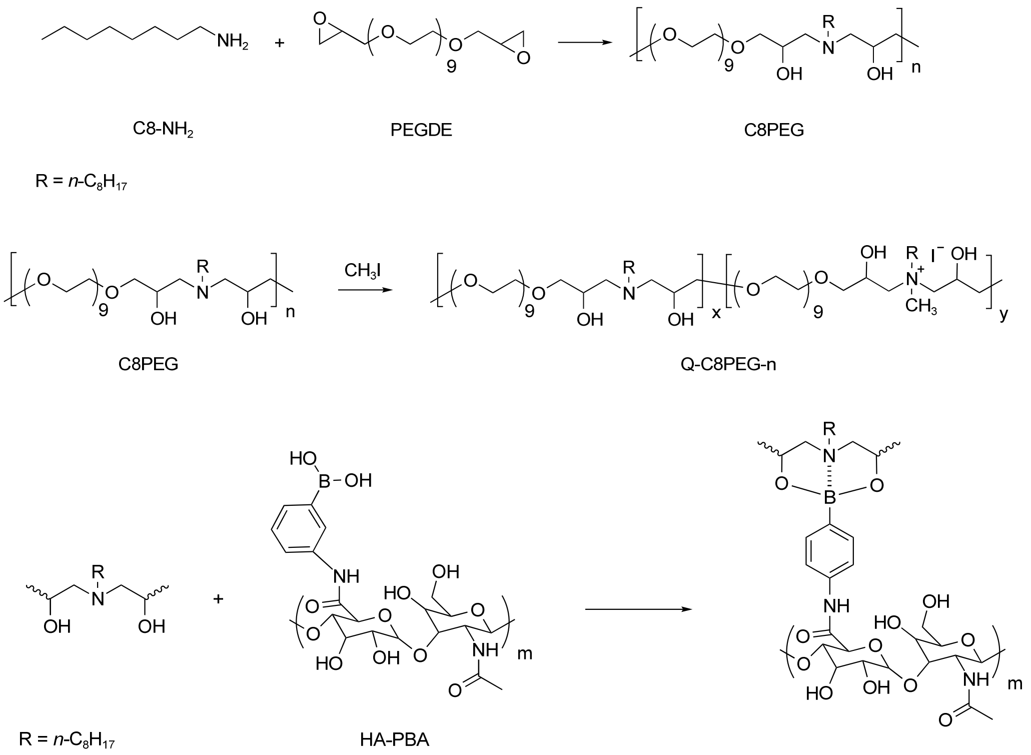

Figure S1. C8PEG and Q-C8PEG-n copolymers synthesis and the gelation with HA-PBA.

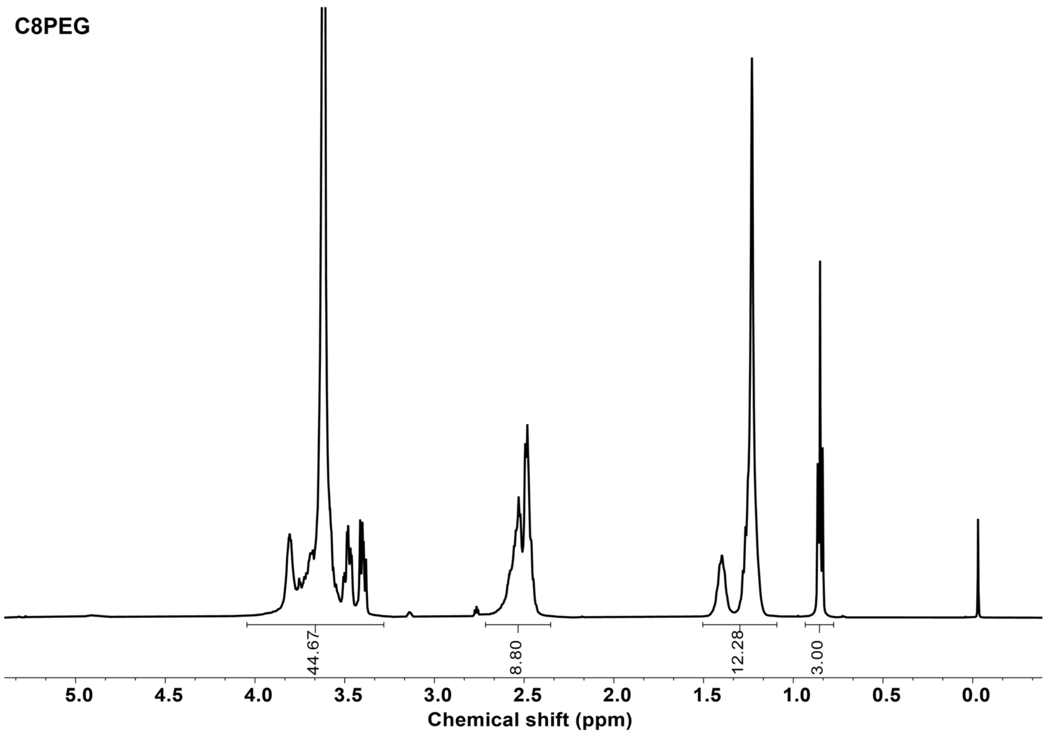

Figure S2. ^{1}H NMR spectrum of C8PEG in $CDCl_3$

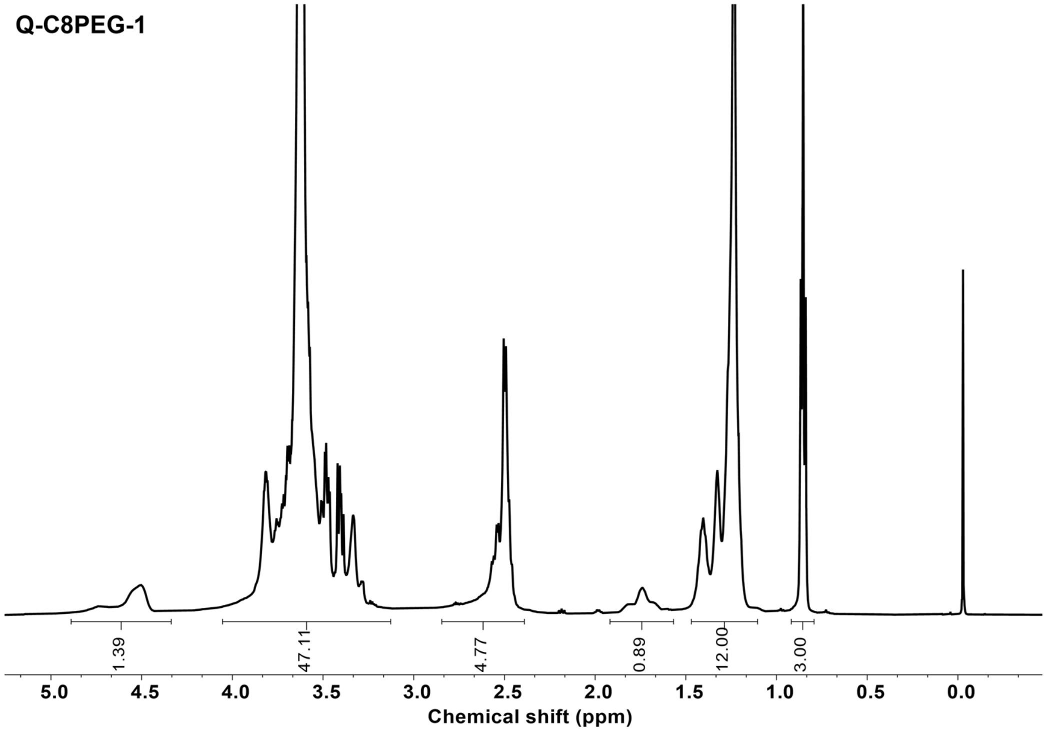

Figure S3. ^{1}H NMR spectrum of Q-C8PEG-1 in $CDCl_3$

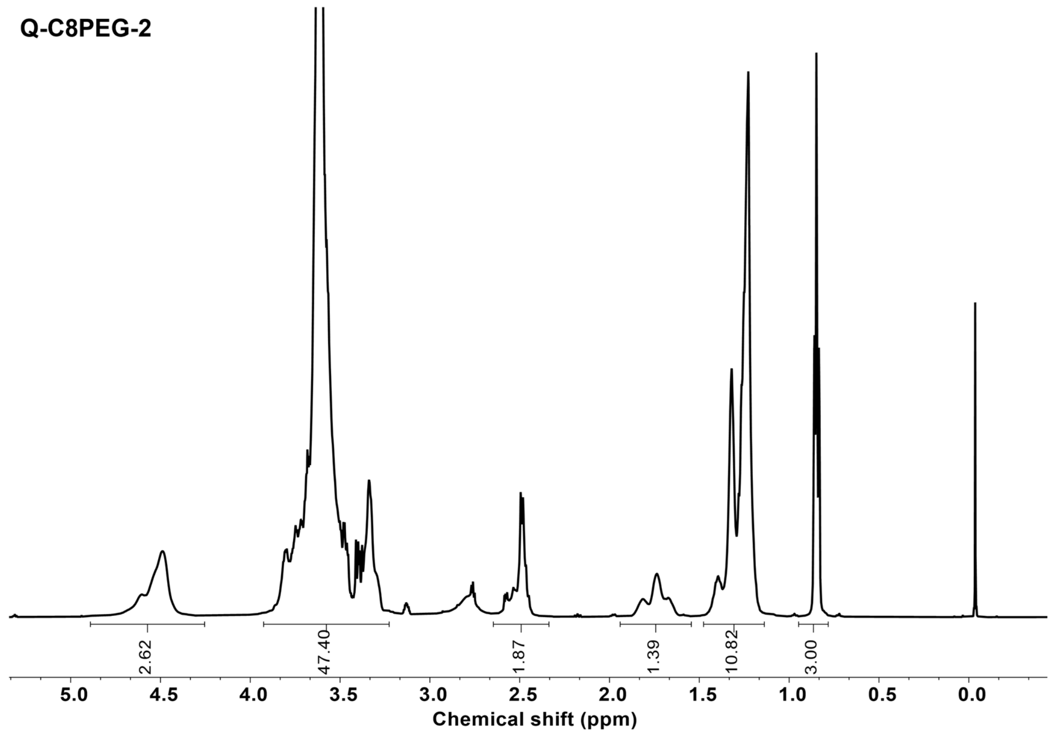

Figure S4. ^{1}H NMR spectrum of Q-C8PEG-2 in $CDCl_3$

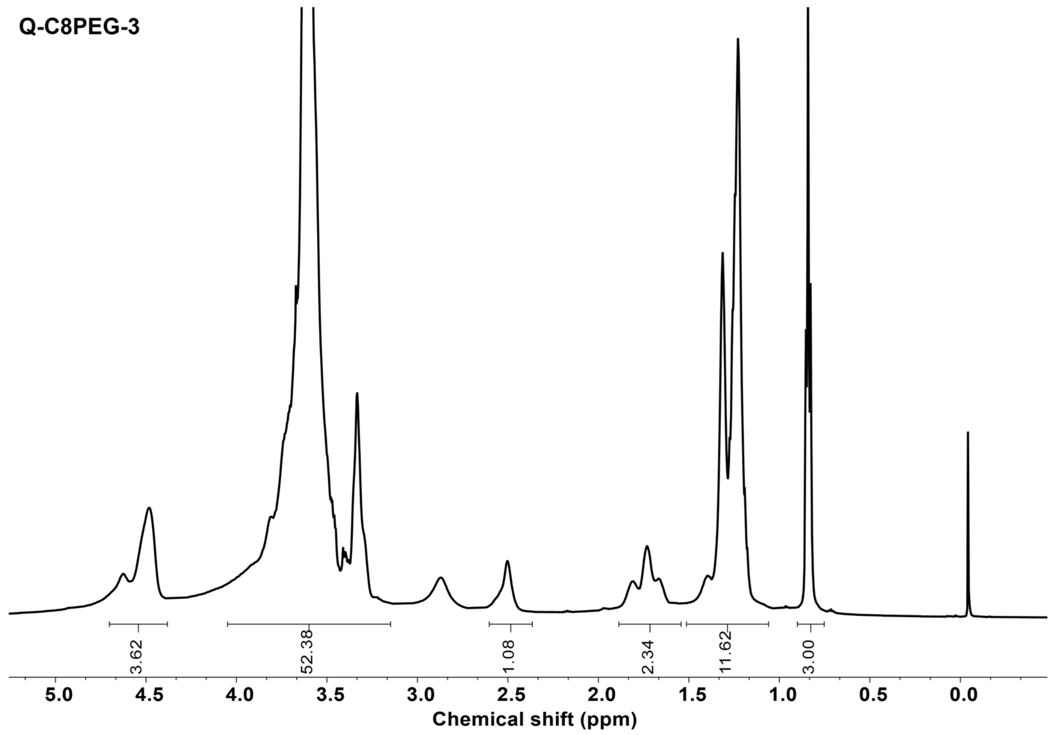

Figure S5. ^{1}H NMR spectrum of Q-C8PEG-3 in $CDCl_3$.

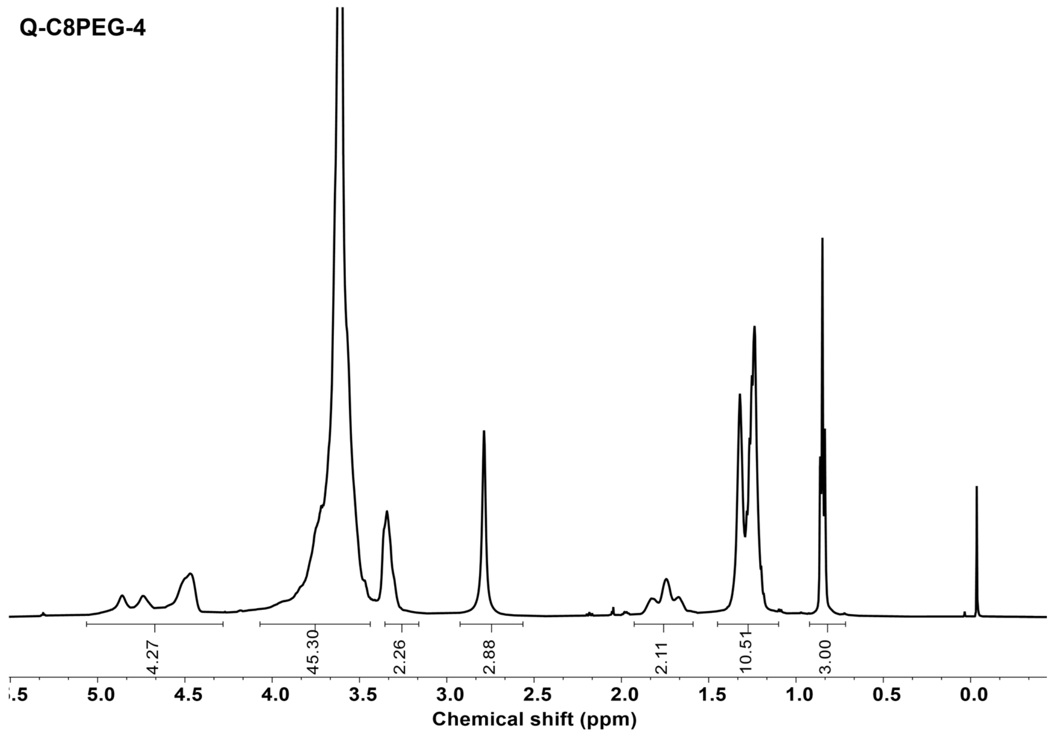

Figure S6. ^{1}H NMR spectrum of Q-C8PEG-4 in $CDCl_3$.

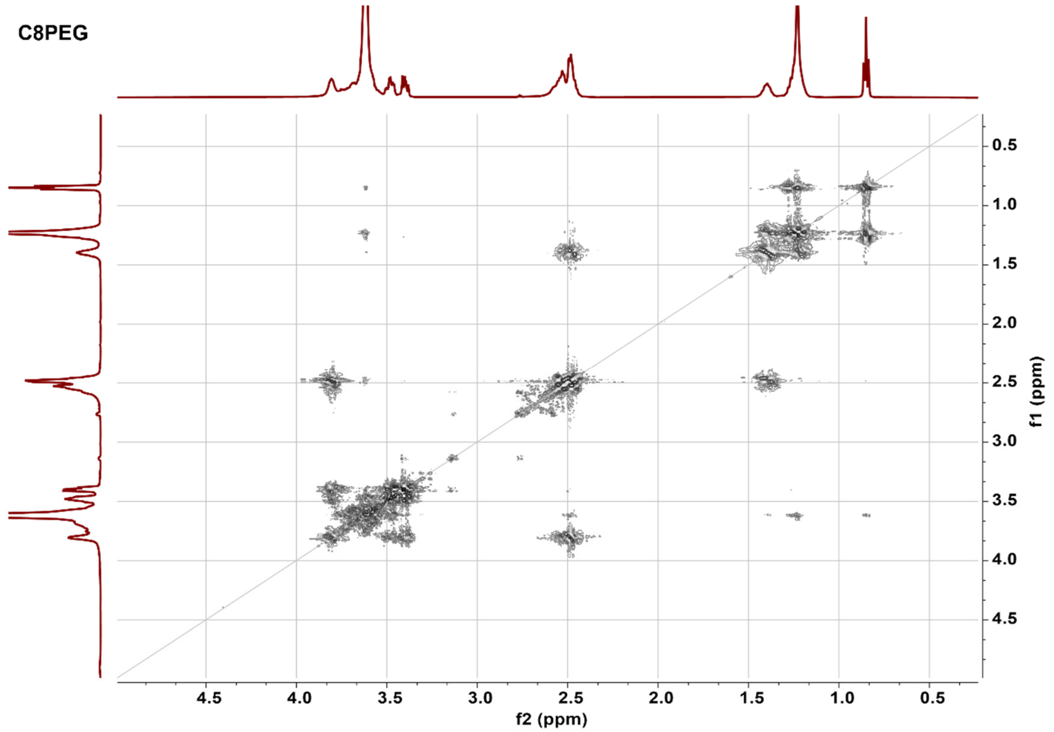

Figure S7. ^{1}H- ^{1}H NMR COSY spectrum of C8PEG in $CDCl_3$.

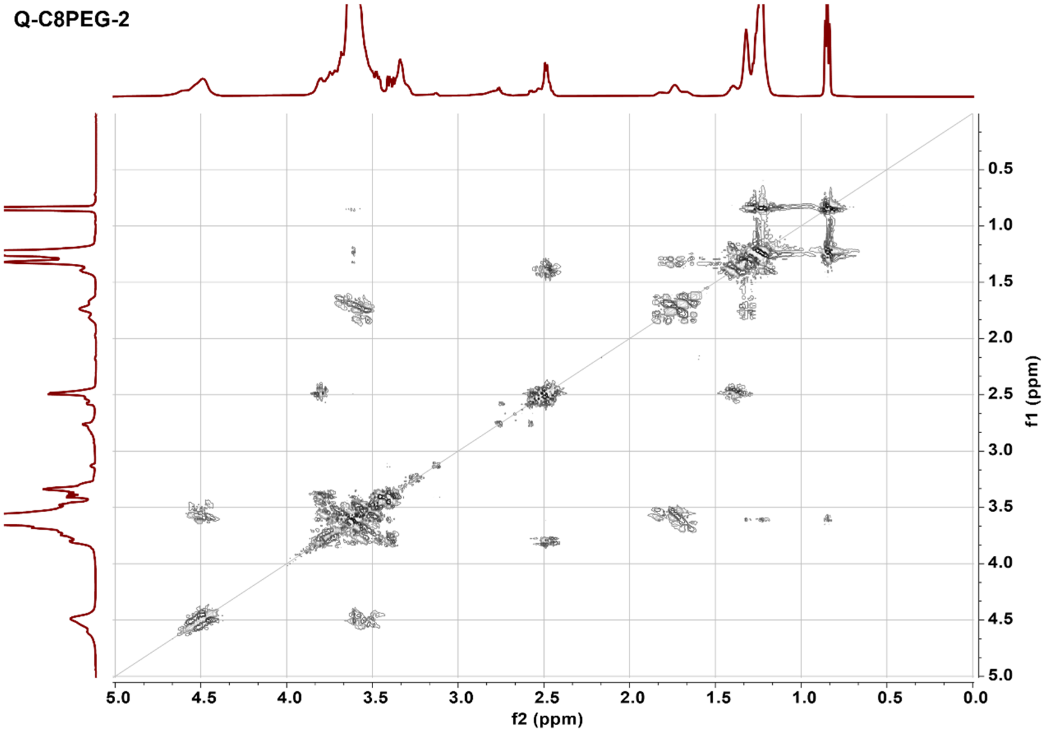

Figure S8. ^{1}H- ^{1}H NMR COSY spectrum of Q-C8PEG-2 in $CDCl_3$.

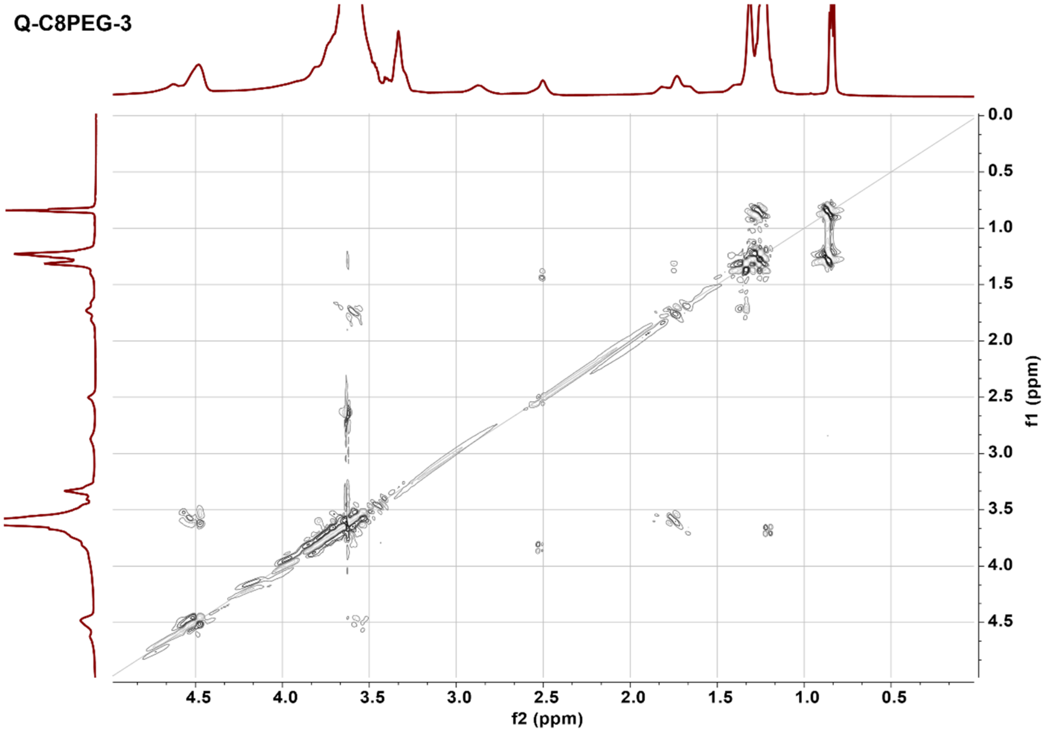

Figure S9. ^{1}H- ^{1}H NMR COSY spectrum of Q-C8PEG-3 in $CDCl_3$.

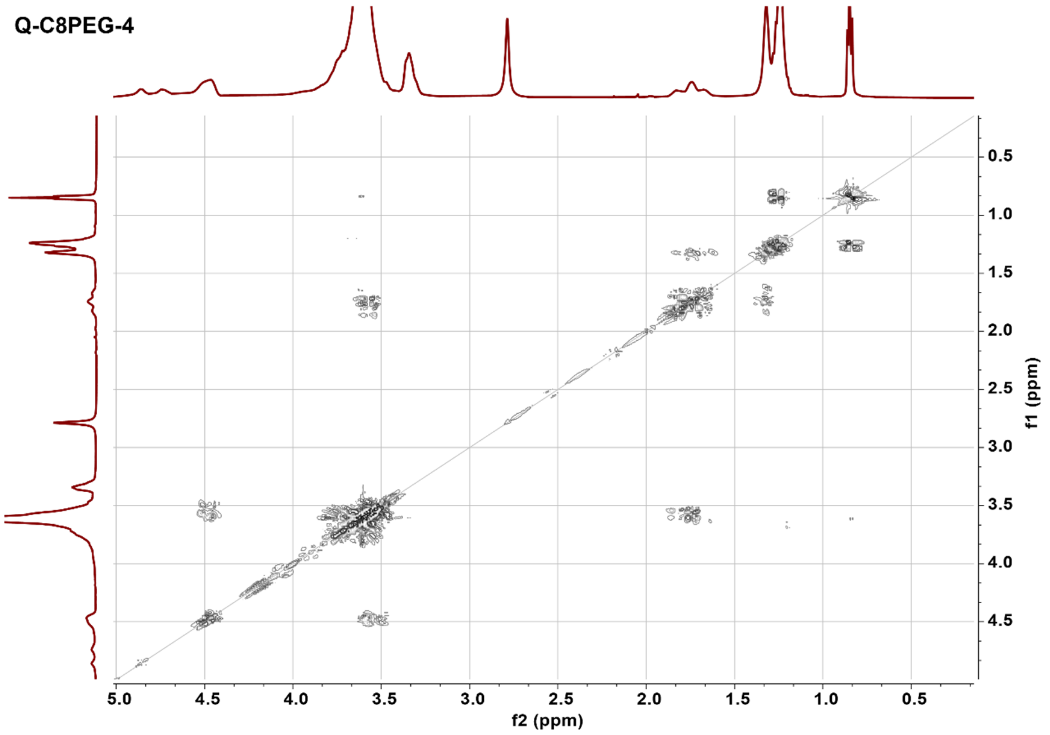

Figure S10. ^{1}H- ^{1}H NMR COSY spectrum of Q-C8PEG-4 in $CDCl_3$.

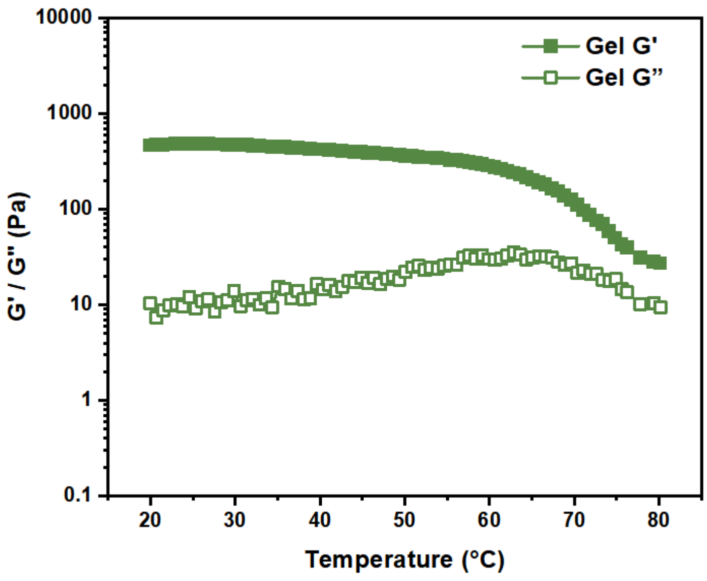

Figure S11. Temperature sweep of Gel from 20 °C to 80 °C.

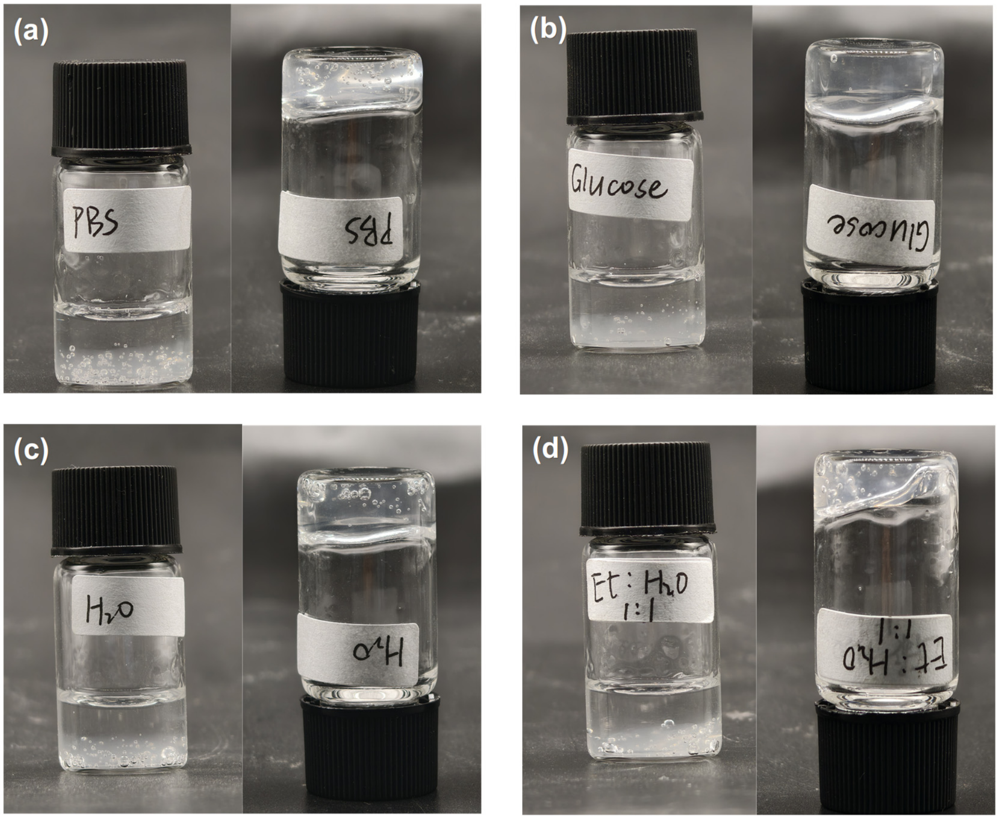

Figure S12 The stability assessment of hydrogels in 1 mL (a) PBS solution, (b) glucose, (c) H_2O, and (d) ethanol-water mixtures.
